# Prophylactic antibiotics to reduce pelvic infection in women having miscarriage surgery – The AIMS (Antibiotics in Miscarriage Surgery) trial: study protocol for a randomized controlled trial

**DOI:** 10.1186/s13063-018-2598-3

**Published:** 2018-04-23

**Authors:** David Lissauer, Amie Wilson, Jane Daniels, Lee Middleton, Jon Bishop, Catherine Hewitt, Abi Merriel, Andrew Weeks, Chisale Mhango, Ronald Mataya, Frank Taulo, Theresa Ngalawesa, Agatha Chirwa, Colleta Mphasa, Tayamika Tambala, Grace Chiudzu, Caroline Mwalwanda, Agnes Mboma, Rahat Qureshi, Iffat Ahmed, Humera Ismail, Metin Gulmezoglu, Olufemi T. Oladapo, Godfrey Mbaruku, Jerome Chibwana, Grace Watts, Beatus Simon, James Ditai, Charles Otim Tom, Jane-Frances Acam, John Ekunait, Helen Uniza, Margaret Iyaku, Margaret Anyango, Javier Zamora, Tracy Roberts, Ilias Goranitis, Nicola Desmond, Arri Coomarasamy

**Affiliations:** 1 Institute of Metabolism and Systems Research, University of Birmingham, Birmingham, B15 2TT UK; 2 Institute of Applied Health Research, University of Birmingham, Birmingham, B15 2TT UK; 3 School of Social and Community Medicine, University of Bristol, Bristol, BS8 2PS UK; 4 Institute of Translational Medicine, University of Liverpool, Liverpool, L69 3BX UK; 5 Department of Obstetrics and Gynaecology, College of Medicine, Blantyre, Malawi; 6 Kamuzu Central Hospital, Lilongwe, Malawi; 7 The Aga Khan University Hospital and Medical College Foundation, Karachi, Pakistan; 8 UNDP/UNFPA/UNICEF/WHO/World Bank Special Programme of Research, Development and Research Training in Human Reproduction (HRP), Department of Reproductive Health and Research, World Health Organization, Geneva, Switzerland; 9 Ifakara Health Institute, Dar es Salaam, Tanzania; 10 Sanyu Africa Research Institute (SAfRI), Mbale, Uganda; 11 Soroti Regional Referral Hospital, Soroti, Uganda; 12 Clinical Biostatistics Unit, Hospital Universitario Ramón y Cajal, CIBER en Epidemiología y Salud Pública (CIBERESP) and Instituto de Investigación Sanitaria (IRYCIS), Madrid, Spain; 13 Health Economics Unit, University of Birmingham, Birmingham, B15 2TT UK; 14 Malawi Liverpool Wellcome Trust, Chichiri, Blantyre, Malawi; 15 Faculty of Medicine & Health Sciences, University of Nottingham, Nottingham, NG7 2RD UK

**Keywords:** Miscarriage, miscarriage surgery, pelvic infection, antibiotics, placebo-controlled trial, randomised controlled trial, low-income countries, economic evaluation

## Abstract

**Background:**

The estimated annual global burden of miscarriage is 33 million out of 210 million pregnancies. Many women undergoing miscarriage have surgery to remove pregnancy tissues, resulting in miscarriage surgery being one of the most common operations performed in hospitals in low-income countries. Infection is a serious consequence and can result in serious illness and death. In low-income settings, the infection rate following miscarriage surgery has been reported to be high.

Good quality evidence on the use of prophylactic antibiotics for surgical miscarriage management is not available. Given that miscarriage surgery is common, and infective complications are frequent and serious, prophylactic antibiotics may offer a simple and affordable intervention to improve outcomes.

**Methods:**

Eligible patients will be approached once the diagnosis of miscarriage has been made according to local practice. Once informed consent has been given, participants will be randomly allocated using a secure internet facility (1:1 ratio) to a single dose of oral doxycycline (400 mg) and metronidazole (400 mg) or placebo. Allocation will be concealed to both the patient and the healthcare providers. A total of 3400 women will be randomised, 1700 in each arm. The medication will be given approximately 2 hours before surgery, which will be provided according to local practice. The primary outcome is pelvic infection 2 weeks after surgery. Women will be invited to the hospital for a clinical assessment at 2 weeks. Secondary outcomes include overall antibiotic use, individual components of the primary outcome, death, hospital admission, unplanned consultations, blood transfusion, vomiting, diarrhoea, adverse events, anaphylaxis and allergy, duration of clinical symptoms, and days before return to usual activities. An economic evaluation will be performed to determine if prophylactic antibiotics are cost-effective.

**Discussion:**

This trial will assess whether a single dose of doxycycline (400 mg) and metronidazole (400 mg) taken orally 2 hours before miscarriage surgery can reduce the incidence of pelvic infection in women up to 2 weeks after miscarriage surgery.

**Trial registration:**

Registered with the ISRCTN (international standard randomised controlled trial number) registry: ISRCTN 97143849. (Registered on April 17, 2013).

**Electronic supplementary material:**

The online version of this article (10.1186/s13063-018-2598-3) contains supplementary material, which is available to authorized users.

## Background

The estimated annual global burden of miscarriage is 33 million out of 210 million pregnancies [1, 2]. The majority of women with a miscarriage will undergo surgery, performed to remove pregnancy tissues from the uterus [3]. In many hospitals in low-income countries, miscarriage surgery is one of the most common operations performed; for example, at Queen Elizabeth Central Hospital, Malawi, an average of 156 suction evacuations for miscarriage are carried out each month, representing 68% of gynaecology admissions [4].

Infection is a serious consequence of miscarriage surgery. The data available from high-income countries found that it occurred in up to 6% of women following surgical management of miscarriage [5, 6]. In low-income settings, the infection rate after miscarriage surgery has been reported to be up to 30% [7]. Infection after miscarriage can result in serious illness and death, as well as long-term consequences from pelvic scarring, increased rates of ectopic pregnancy and infertility. Ectopic pregnancies can result in death from severe haemorrhage, and infertility has profound social and economic effects that go beyond childlessness, with women bearing the brunt of the burden [8].

For surgical termination of pregnancy, the standard practice is to provide prophylactic antibiotics before or during surgery as there is evidence of benefit for this intervention [9] and unanimous endorsement from policymakers and practitioners [9–13]. However, evidence is insufficient for miscarriage management and therefore, unsurprisingly, policymakers do not recommend, and practitioners do not consistently provide, prophylactic antibiotics during surgery. However, given that miscarriage surgery is common, and infective complications are frequent and serious, prophylactic antibiotics, if found to be effective in a well-conducted and adequately powered trial, may offer a simple and affordable intervention to reduce the burden of disease in low-income countries.

## Methods/Design

### Aims and objectives

The primary objective is to test the hypothesis that, in women having miscarriage surgery, pre-surgery prophylactic antibiotics (oral doxycycline 400 mg and oral metronidazole 400 mg) reduce the risk of pelvic infection within 14 days of surgery. In cases where participants do not return for follow-up within this period, follow-up until 28 days will be acceptable.

The secondary objectives are to test whether prophylactic antibiotics result in a reduction in maternal mortality, duration of clinical symptoms, hospital admission, unplanned consultations and overall antibiotic use. We will also examine the individual components of the primary outcome and test the hypothesis that prophylactic antibiotics, compared to placebo, do not incur serious adverse effects to the mother.

We will explore differential effects of antibiotic prophylaxis in the subgroups of (1) type of surgery (manual vacuum aspiration, suction curettage or sharp curettage), (2) type of miscarriage (incomplete or missed), (3) gestational age (< 12 weeks or ≥ 12 weeks, when known), (4) HIV status (positive or negative, when results available), (5) time between administration of antibiotics and surgery, (6) country and sites, and (7) rural or urban residence. In addition, we will perform an economic evaluation to determine if prophylactic antibiotics are cost-effective.

### Design and setting

The AIMS trial is a randomised, double blind, placebo-controlled multicentre study, with a health economic evaluation. The trial is set in a diverse range of hospitals across four countries, namely in Malawi at Queen Elizabeth Central Hospital, Zomba Central Hospital and Kamuzu Central Hospital; in Uganda at Mbale Regional Referral Hospital and Soroti Regional Referral Hospital; in Tanzania at Bagamoyo District Hospital, Mwananyamala Hospital and St Francis Hospital; and in Pakistan at Aga Khan University Main Hospital, Kharader Hospital, Garden Hospital, Hyderabad Hospital and Karimabad Hospital.

These participating countries were selected as this is a clinical problem that is of particular importance in low- and middle-income countries. Final country selection was based on the need for generalisability of the findings, prevalence of miscarriage surgery in these countries, importance of the clinical question in the participating country, existing research links, and research infrastructure to appropriately deliver the study to a high quality.

### Participants

**Fig. 1:**
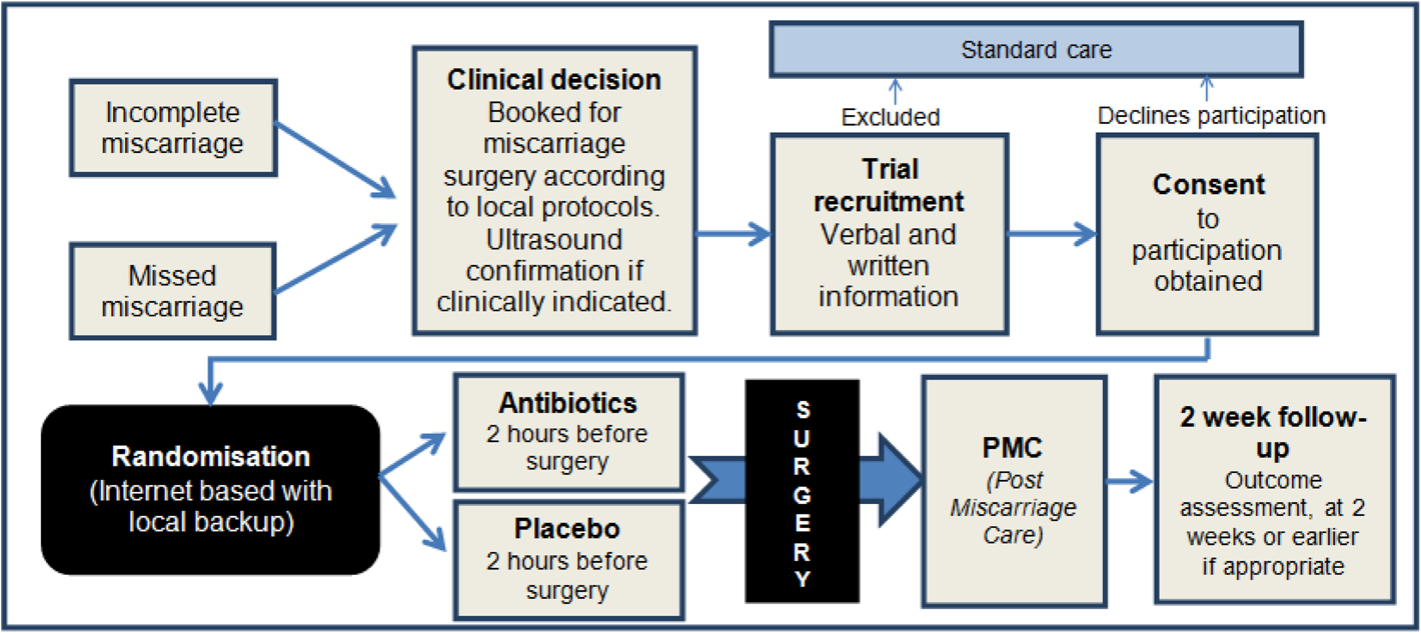
Participant flow through trial

The AIMS trial will include women with a spontaneous miscarriage under 22 weeks gestation undergoing surgical evacuation of the uterus (by manual vacuum aspiration, suction curettage or sharp curettage) that are willing and able to give informed consent, and are undergoing their first surgical evacuation in the current pregnancy (Fig. 1). Women having induced abortion of pregnancy, or with a septic miscarriage, evidence of infection or current febrile illness (temperature above 38 ºC) will be excluded. Women with an allergy or other contraindication to either of the antibiotics, or using antibiotics currently or within the 7 days preceding surgical evacuation, will also be excluded, as will women with conditions requiring immediate care, e.g. severe haemorrhage, women under 16 years of age or women requiring further surgical evacuation after the first surgical evacuation for the current pregnancy (multiple evacuations for the same pregnancy will not be included).

Potentially eligible patients will be approached in an adequate setting (i.e. outpatient clinics, wards, clinical assessment areas) and informed about the trial by an appropriately trained researcher or care provider. After all eligibility criteria has been confirmed, consent has been obtained and all baseline data gathered, participants will be randomised online via a secure internet facility to enable the allocation to remain concealed to the research staff and the care providers, as well as the participants, in a 1:1 ratio through an Integrated Trial Management System, to the investigational medicinal product or the placebo. Unblinding will only be possible by contacting the clinical trials unit. A minimisation procedure using a computer-based algorithm will be used to avoid chance imbalances in important stratification variables. Strata used in the minimisation will be age (below 35, or 35 years and above), gestation (below 12 weeks, or 12 weeks and above, or when the gestation is unclear), the type of miscarriage (either incomplete or missed), and HIV status (positive, proven negative, or unknown).

### Study intervention and comparison

The investigational medicinal products (IMPs) are doxycycline, as four 100 mg doxycycline hyclate encapsulated tablets, and metronidazole, as one 400 mg encapsulated tablet [14, 15]. The placebo tablets are encapsulated microcrystalline cellulose in the same format as the IMPs, identical in colour, shape and weight, produced and distributed by Sharp Clinical UK. Both healthcare provider and participant will be blinded to the allocation. The study medications will be taken approximately 2 hours before surgery. The IMP will be administered, with consent, under the direct observation of the clinician or researcher consenting the patient for the trial. Therefore, we do not anticipate compliance to be an issue unless the patient choses to withdraw their consent between randomisation and the IMP being taken. The choice of antibiotics was made after a careful review of the existing literature, a survey of preference, availability and affordability in the partner countries, and a review of other related evidence from induced abortion guidelines. Doxycycline is active against a broad range of Gram-positive and Gram-negative aerobic bacteria, including staphylococci, streptococci, and enteric Gram-negative bacteria. It is also active against *Chlamydia trachomatis* and *Neisseria gonorrhoeae*. Metronidazole is effective against anaerobic bacteria.

Doxycycline is associated with a substantially lower risk of life-threatening anaphylaxis compared with co-amoxiclav. Both of the study medicines are commonly used in low-income settings, and have low adverse event profiles. There is an extremely low risk of anaphylaxis, which was one of the reasons for the choice of these agents. However, should an anaphylactic reaction occur, all sites have facilities to manage this.

The use of other non-trial medications is at the discretion of the care-providing clinicians. Unblinding will only be performed in the event of a medical emergency in which treatment of the emergency requires knowledge of the actual drug received.

### Follow-up

Trial participants will be given multiple easy access routes to contact the research and clinical teams to report issues and seek advice and care. Women contacting the study or research team will be reviewed promptly. Reimbursement for potential telephone and transport costs will be offered to participants. Non-attendance at follow-up clinics will prompt telephone calls or others means of contacting the participant, which will be agreed at recruitment. If possible, contact will be made with the participant prior to the clinic to remind them through, for example, telephone calls or SMS text messages to prompt the reporting of any infection or other adverse events and follow-up clinic attendance.

### Withdrawal from study

A participant can be withdrawn from the trial if, in the opinion of the investigator, it is medically necessary. With premature withdrawal from the study, the study personnel will make every effort to obtain, and record, information about the reasons for discontinuation and any adverse events and to perform all safety assessments. A patient may voluntarily withdraw from participation in this study at any time. If the patient does not return for a scheduled visit, we will try to contact her. We will aim to document the reason for withdrawal and, when possible, all safety assessments will be performed. If a patient explicitly withdraws consent to have data recorded, their decision will be respected and recorded on the trial database. All communication surrounding the withdrawal will be noted in the patient’s records and no further data will be completed for that patient.

### Statistical analysis

The primary outcome is pelvic infection within 14 days of surgery. At the commencement of the study pelvic infection was defined (based on the CDC criteria and criteria used in previous studies) as two or more of (1) purulent vaginal discharge, (2) pyrexia > 38.0 °C, (4) uterine tenderness on examination and (4) a white cell count > 12 × 10^9^/L [14], with no other recognised cause of infection upon history taking and examination by the assessing clinician and/or study team (Table 1).

**Table 1:** Primary outcome measure ascertainment

	Purulent vaginal discharge	Pyrexia	Tenderness	White cell count
Definition	Yellow, green, or offensive discharge	> 38.0 °C measured by infrared ear thermometry	Defined as a finding on examination of either (1) direct tenderness of the uterus, or (2) tenderness on displacement of the uterus, or (3) tenderness of the parametrium or (4) tender adnexal mass	White cell count greater than 12 × 10^9^/L
Excluded from the definition	Clear vaginal discharge		Upper abdominal pain	

An amendment was made to these criteria, after recruitment commencement, but without reference to study data, as it was observed by the examining clinicians (who were blinded to treatment allocation) that, for some participants, whilst only a single feature of pelvic infection was present, the symptoms were of sufficient severity that, in their clinical judgement, there was a need to provide treatment. After discussion with the independent trial steering committee it was decided that the diagnostic criteria by which pelvic infection was defined should be adjusted. Following this amendment, pelvic infection was diagnosed if the patient had two or more of (1) purulent vaginal discharge, (2) pyrexia > 38.0 °C, (3) uterine tenderness on examination and (4) a white cell count > 12 × 10^9^/L [16], with no other recognised cause of infection upon history taking and examination by the assessing clinician and/or study team, or one of the above features, with a clinically identified need to administer antibiotics for the treatment of presumed pelvic infection (Table 1: primary outcome measure ascertainment). The latter pragmatic criteria will be reported as the primary outcome, but the more specific strict clinical criteria for diagnosis of pelvic infection will also be clearly reported.

In cases where participants do not return for follow-up within the specified period of 2 weeks, follow-up until 28 days will be acceptable.

The secondary outcomes are overall general antibiotic use (not specifically for pelvic infection), each component of the primary outcome, death, hospital admission, unplanned consultations, blood transfusion, vomiting, diarrhoea, adverse events, anaphylaxis and allergy, duration of clinical symptoms (pain, additional analgesia, vaginal bleeding), and days before return to usual daily activities.

We are proactively seeking evidence of adverse events. Each participant will be asked, at each trial visit or interview, about hospitalisations, consultations with other medical practitioners, disability or incapacity, or any other adverse events.

The analysis will be by intention-to-treat. The risk ratio of women with pelvic infection in the active arm to the placebo arm will be calculated. This estimate will be adjusted for the minimisation variables although an unadjusted estimate will also be calculated as a sensitivity analysis.

In the first instance, analysis will be completed on received data only, with every attempt made to gather data on all subjects randomised, irrespective of compliance with the treatment protocol. To test the robustness of this initial analysis with respect to any missing data, missing responses will be simulated using a multiple imputation approach as a sensitivity analysis. Further sensitivity analysis will be performed assuming all non-responders had a pelvic infection.

Seven pre-specified subgroup analyses are planned to assess differential effects of antibiotic prophylaxis in (1) type of surgery (manual vacuum aspiration, suction curettage or sharp curettage; sharp curettage is associated with a higher incidence of pelvic infection when compared to suction curettage [3], so a greater effect of antibiotic prophylaxis may be seen in the sharp curettage group); (2) type of miscarriage (incomplete or missed; incomplete miscarriage may be associated with a higher incidence of pelvic infection when compared to missed miscarriage [3]); (3) gestational age (< 12 weeks or ≥ 12 weeks, when known; pregnancies of a greater gestational age may have a higher incidence of pelvic infection due to the additional complexities involved with the surgical procedure [3]); (4) HIV status (positive or negative, when results are available; participants that are HIV positive may be more likely to experience pelvic infection as they may be immunocompromised); (5) by time between administration of antibiotics and surgery (a reduced time interval between antibiotic administration and surgery, than that specified in the protocol, may result in a reduction in bio-availability and thus in effect reduction; the optimal maximum duration however is unclear); (6) by country and sites; and (7) rural or urban residence (the potential direction of effect is unclear in sub-group analyses (6) and (7)).

We will randomise 3400 women in total, which will give 90% power to detect a relative risk reduction of 0.4 (an RR of 0.6) (type I error rate, *P* = 0.05). A meta-analysis of 18 studies of prophylactic antibiotics used for induced abortion surgery was conducted to inform these calculations and found a pooled RR of 0.60 (95% CI 0.48–0.74) for the reduction of pelvic infection. We calculated sample sizes to detect this size of difference using various scenarios for the baseline risk (varying from 3% to 10%). The best available data for pelvic infection rates specific to spontaneous miscarriage originate from studies in the UK and USA, demonstrating a risk of pelvic infection of up to 6%; the risk of pelvic infection is likely to be higher in low-income countries, yet even for a baseline risk of, the 5% sample size of 3400 will provide over 80% power to detect a difference in primary outcome. Further sensitivity analysis will be performed assuming all non-responders had a pelvic infection.

Interim analyses of principal safety and effectiveness endpoints will be conducted on behalf of an independent Data Monitoring Committee (DMC). These will be considered together with a report of serious adverse events. A pragmatic approach to stopping rules was suggested by the DMC members with respect to interpreting the results of any interim analysis. Appropriate criteria of proof will therefore not be specified precisely, but an interim analysis difference equating to *P* < 0.001 (similar to a Haybittle–Peto boundary) may be needed to justify halting, or modifying, of the study prematurely. This criterion has the practical advantage that the exact number of interim analyses would be of little importance and there would only be minimal inflation of any overall type-I error rate regardless of how many interim analyses are performed, so no exact schedule needs to be proposed.

### Health economic evaluation

The economic evaluation will determine the relative cost-effectiveness of antibiotic prophylaxis in the surgical management of miscarriage compared to the current practice without antibiotic prophylaxis. All resource use data will be prospectively collected using case report forms completed on at least one occasion prior to discharge after surgery, at every contact during the follow-up period, daily whilst an inpatient, and at final assessment. Resource use information will be employed to estimate the costs associated with the additional use of antibiotics in all participating centres. This will include antibiotics and other medications related to pain, allergy, diarrhoea, vomiting, nausea, malaria and fever as well as inpatient stays, outpatient visits, laboratory examinations and treatment of relevant complications, such as haemorrhage requiring blood transfusion, repeat uterine evacuation and anaphylaxis. Unit cost data will be obtained from the International Drug Price Indicator Guide [17], the World Health Organization (WHO-CHOICE) [16] and other secondary sources, and will be adjusted to 2016 US Dollars. Given the short time horizon, no discounting will be applied to costs. An incremental cost-effectiveness analysis will be conducted from a healthcare provider perspective based on the outcome of cost per pelvic infection avoided within 2 weeks from surgery using established health economic methods for multinational trials [18, 19]. The uncertainty around cost-effectiveness point estimates will be presented using a cost-effectiveness acceptability frontier [20]. Sensitivity analyses will be performed to explore the robustness of the study findings to plausible variations in key values.

### Conduct and monitoring of the trial

The Birmingham Clinical Trials Unit is responsible for the coordination of the trial; it is a fully registered UK Clinical Research Collaboration clinical trials unit, and provides a robust quality management system to ensure good practice in the conduct and statistical analysis of the project. The trial is being conducted in accordance with the ethical principles that have their origin in the Declaration of Helsinki, 1996, namely the principles of Good Clinical Practice. The trial was not initiated at each site before full approval from the UK Research Ethics Committee, and the respective national Research Ethics Committee and local regulatory bodies was received. All AIMS investigators are responsible for (1) maintaining the protocol of the trial as described in this document, (2) helping healthcare professionals to ensure the study participants receive appropriate care throughout the period of research enrolment, (3) protecting the integrity and confidentiality of clinical and other records and data that may be generated by the research, and (4) reporting any failures in these respects, adverse drug reactions and other events or suspected misconduct through the appropriate systems (Additional file 1).

Accrual is monitored against set targets. If a centre is not meeting its recruitment targets then the Trial Management Group will work with the local Principal Investigator and team to identify barriers to recruitment and solutions to any problems. Communication between the centres will enable the spread of good practice and experience between sites. Follow-up requirements are carefully explained to each participant. Written information is also provided in the form of the “follow-up card” (Additional file 3). Trial participants are given multiple easy access routes to contact the research and clinical teams to report issues and seek advice and care. Women contacting the study or research team are reviewed promptly. Reimbursement for potential telephone and transport costs are offered to participants. Non-attendance at follow-up clinics prompts telephone calls or other means of contacting the participant, which are agreed at recruitment. If possible, contact is made with the participant prior to the clinic to provide them a reminder through, for example, telephone calls or SMS text messages to prompt the reporting of any infection or other adverse events and follow-up clinic attendance. Details about complaints are provided in the patient information sheet. Management of complaints is the responsibility of the local Principal Investigator and should follow any locally available procedures before reporting to the trial management group.

Collected data are stored on secure computers. The necessary trial data is encrypted. Electronic data is backed up every 24 h to both local and remote media in encrypted format. Paper-based data (e.g. signed consent forms) are kept locked at each site. Individual participant information obtained as a result of this study is considered confidential. Each participant is allocated a unique study number at recruitment. All documents use this as the identifier. All data will be analysed and reported in summary format. No individual will be identifiable.

The Trial Steering Committee provides overall supervision of the trial and ensures that it is being conducted in accordance with the principles of Good Clinical Practice and other relevant regulations. The DMC reviews the accruing trial data and assesses the safety data to make recommendations on whether the trial should continue, be modified or be terminated. In addition, the DMC also examines effectiveness data to determine if continuation of the trial is unethical, and examines the recruitment, loss to follow-up, compliance and protocol violation data to ascertain if continuation of the trial is futile.

## Discussion

If found beneficial, antibiotics will reduce immediate complications such as sepsis, the need for further operations and death, as well as long-term complications such as subfertility, pelvic pain and ectopic pregnancy. Furthermore, if beneficial, their use would result in a reduction in the financial costs borne by women who have had miscarriages, including reduced loss of earnings and reduced treatment, transport and subsistence costs associated with infective complications, and the long-term health consequences such as chronic pelvic pain. Chronic pelvic pain is a recognised complication of infective complications after miscarriage and is known to have a major impact on work productivity.

If found to be of no benefit, then guidance to avoid prescribing in this situation could be strengthened, reducing unnecessary antibiotic usage, risks of allergy and antimicrobial resistance.

This double-blind randomised placebo controlled trial addresses the key clinical question of whether prophylactic doxycycline (400 mg) and metronidazole (400 mg), prior to miscarriage surgery, can prevent pelvic infection. We anticipate that the findings from this definitive, multicountry study, will inform international practice.

## Trial status

The protocol was submitted on April 7, 2017, at which point all countries were still recruiting to the trial.

## Additional files

Additional file 1: Follow-up card. (DOCX 605 kb)

Additional file 2: Consent form. (DOCX 89 kb)

Additional file 3: Patient information sheet. (DOCX 65 kb)
