# Supplementary material for: Prophylactic antibiotics to reduce pelvic infection in women having miscarriage surgery – The AIMS (Antibiotics in Miscarriage Surgery) trial: study protocol for a randomized controlled trial
Source: Trials. 2018 Apr 23;19:245. doi: 10.1186/s13063-018-2598-3 (PMC5914072; doi:10.1186/s13063-018-2598-3)
Supplement: Supplementary file 1 — Follow-up card. (DOCX 605 kb) [file 13063_2018_2598_MOESM1_ESM.docx]

# Additional file 1

# Follow-up card

(A5 size)


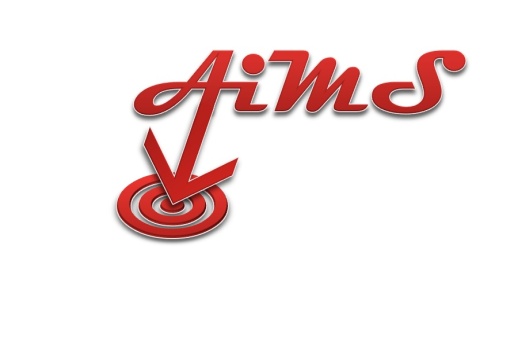


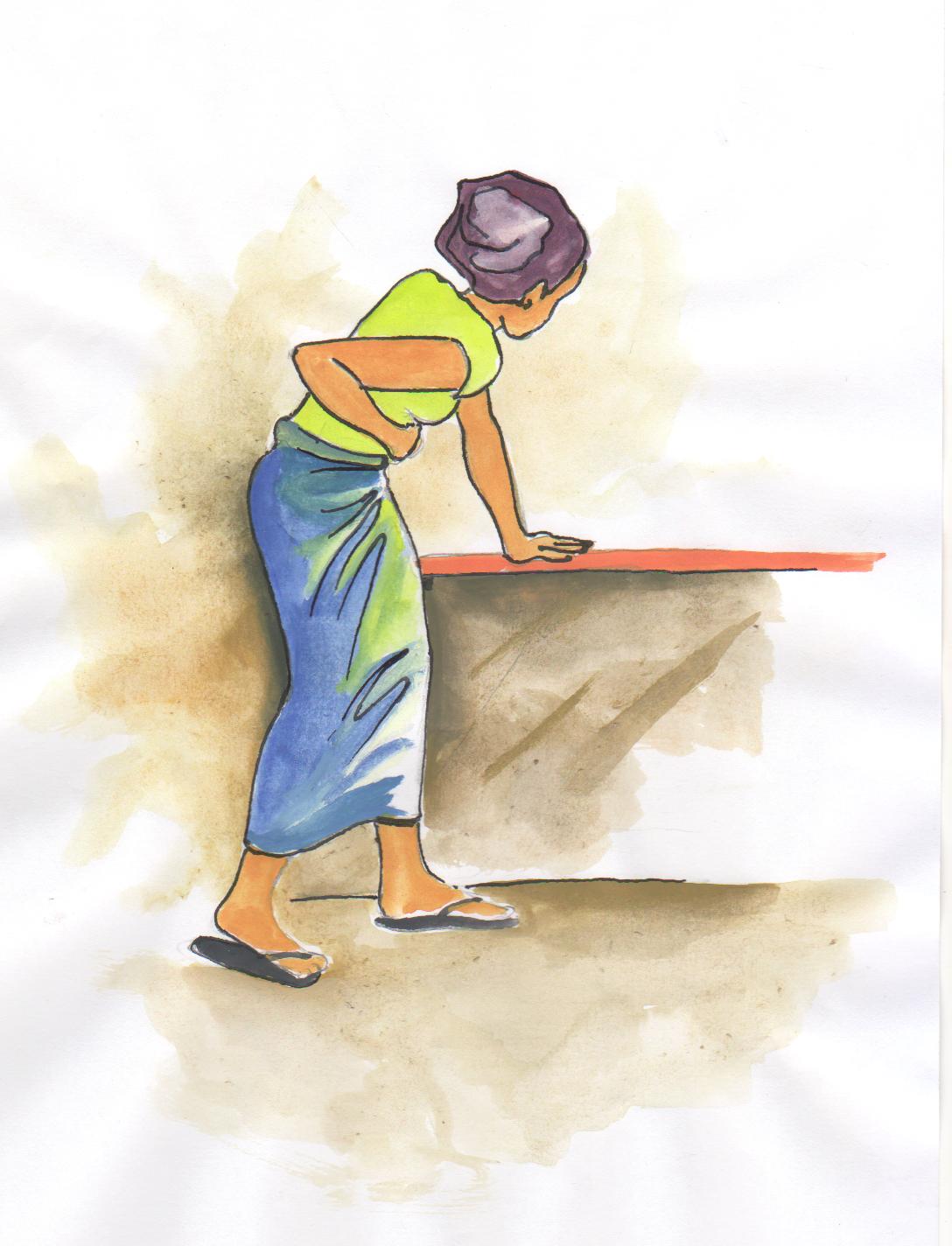


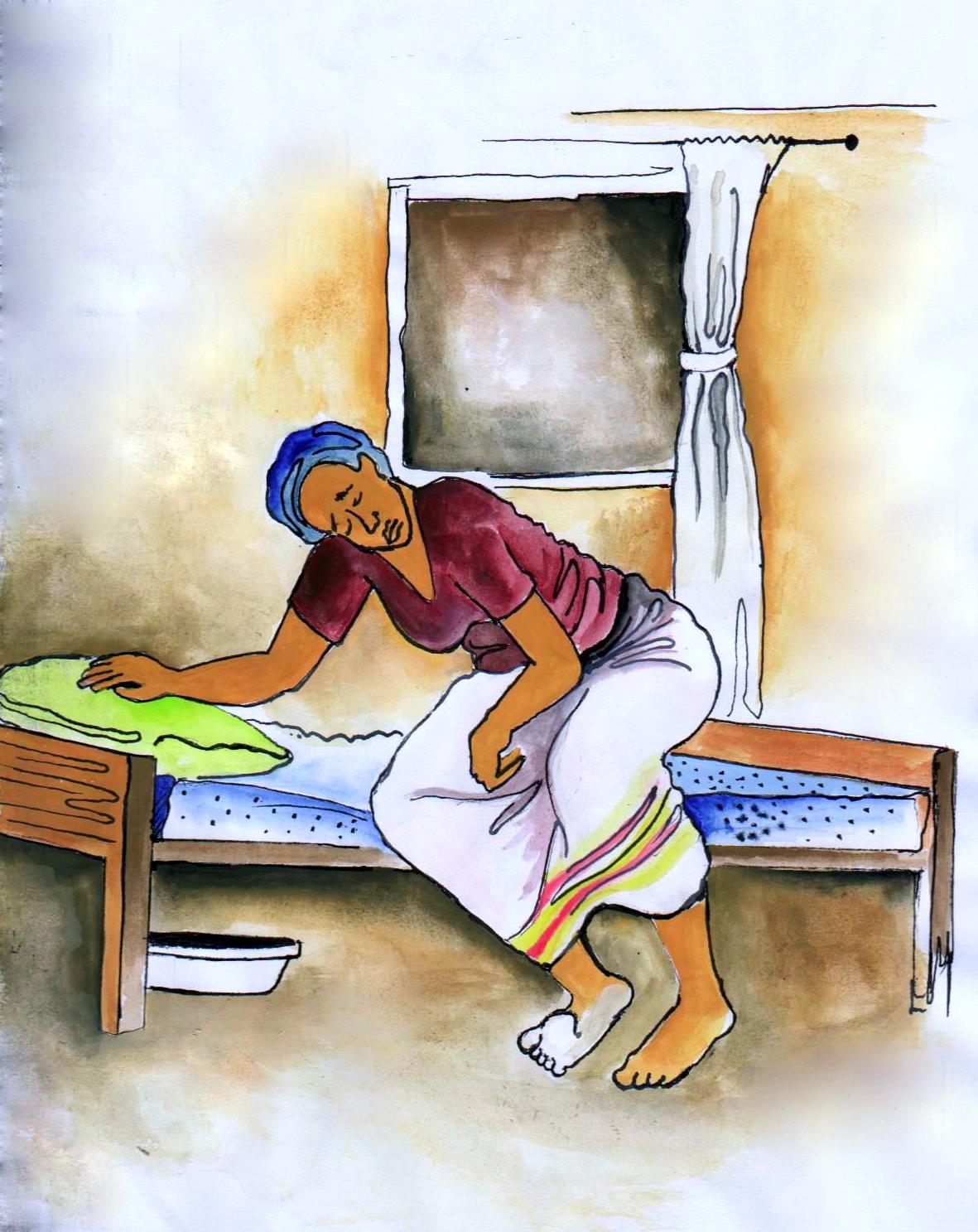


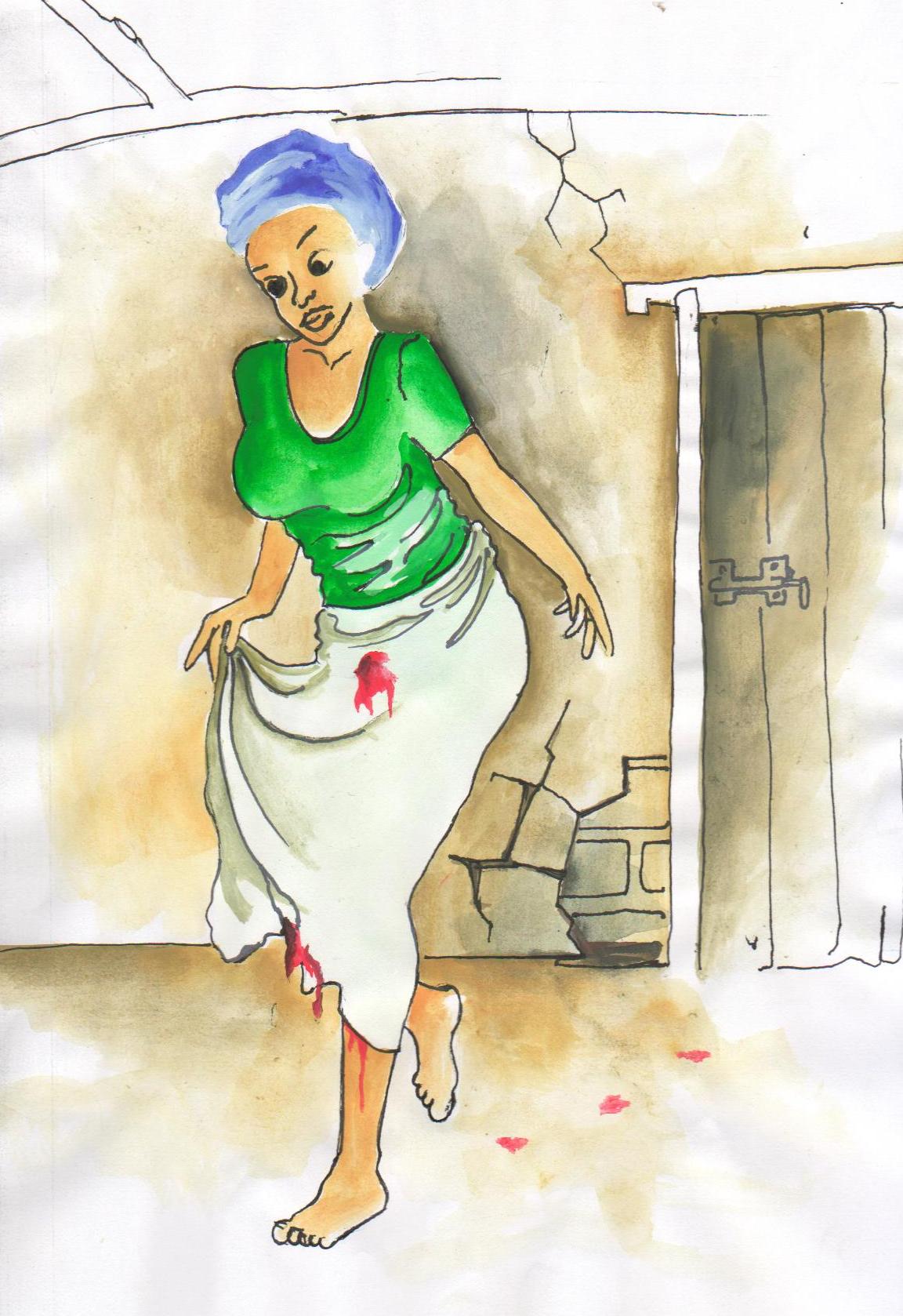


Please contact us immediately if you have any problems in the 14 days after your surgery, or if you have any concerns.

In particular please let us know if you notice any of these things:

- Pain in your lower abdomen
- Feeling feverish, hot and cold or shivery
- Feeling generally unwell
- Any rashes or swelling
- Any abnormal discharge from the vagina
- Any increase in bleeding from the vagina or passing blood clots
- Any other thing that concern you

**A**ntibiotics **I**n **M**iscarriage **S**urgery trial


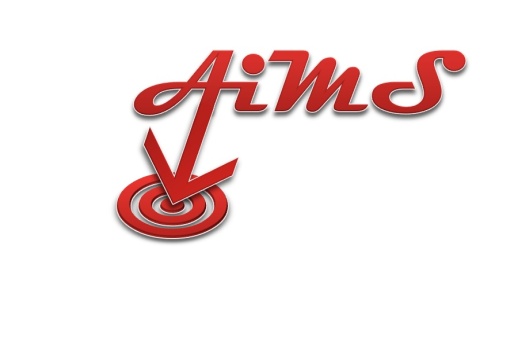


If you experience any health problems in the 14 days (until the ______ of _________) following your miscarriage surgery please let the trial team know.

Date of miscarriage surgery:__________________________

Date and time of follow-up appointment:________________

The follow-up appointment will be at:

Dr [insert name]

[insert clinic] AIMS trial clinic

[insert ward] Ward

[insert hospital] Hospital

You can call the local trial team at any time on [insert number]

You can come to the AIMS trial clinic any day if you want to see a member of the AIMS trial team. The clinic will be open from [insert time] to [insert time], Monday to Friday.

Outside these hours you can go to the Gynaecology ward (Ward [insert ward]) at [insert hospital name] hospital at any time and they will assist you.

Transport costs and phone credit costs will be provided to reimburse you for any expenses and help you to keep contact with us.

**A**ntibiotics **I**n **M**iscarriage **S**urgery trial
