# Supplementary material for: Prophylactic antibiotics to reduce pelvic infection in women having miscarriage surgery – The AIMS (Antibiotics in Miscarriage Surgery) trial: study protocol for a randomized controlled trial
Source: Trials. 2018 Apr 23;19:245. doi: 10.1186/s13063-018-2598-3 (PMC5914072; doi:10.1186/s13063-018-2598-3)
Supplement: Supplementary file 2 — Consent form. (DOCX 89 kb) [file 13063_2018_2598_MOESM2_ESM.docx]

# Additional file 2

# Consent form

| *D* | *D* | *M* | *M* | **2** | **0** | *Y* | *Y* |
| --- | --- | --- | --- | --- | --- | --- | --- |

**ANTIBIOTICS IN MISCARRIAGE SURGERY (Aims) TRIAL**


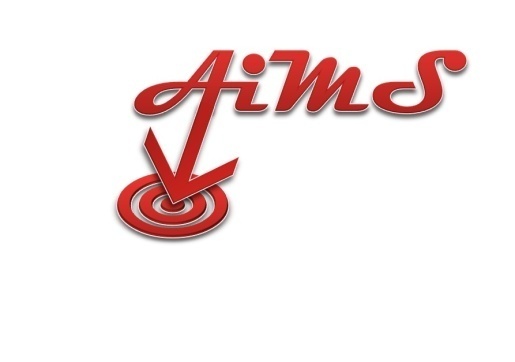


**Trial ID**

**Date of Form**

|  |  |  |  |  |  |  |  |
| --- | --- | --- | --- | --- | --- | --- | --- |

*Please print on a local headed paper with the Local Hospital/Centre details and Logo.*

**Hospital ID**

**Participant Initials**

Please initial the boxes below

Please initial the boxes below

Please initial the boxes below

Please initial the boxes below

**CONSENT FORM**

1. I have read/ or had read to me, the information sheet for the AIMS study (version 1.0, dated 21.03.2013) and have had the opportunity to consider the information, ask questions, and have these answered satisfactorily.
2. I understand that participation in this study is entirely voluntary and that I am free to withdraw at any time, without giving a reason and without my medical care being affected.
3. I understand that my medical notes may be looked at by members of the research team, and by regulatory bodies auditing research practice.
4. I consent to taking part in the AIMS study, which will require me taking the antibiotics or dummy tablet before my miscarriage surgery.
5. I agree to return for a follow-up appointment 2 weeks after my surgery, or before if I become unwell and for the study team to also contact me to gather information about my health after the surgery.
6. I agree to allow the AIMS research team to try and obtain information about my wellbeing from hospital registries or from relatives or friends if the team has not been able to contact me directly for more than 2 weeks
7. I agree to a sticker describing my involvement in the study to be placed in my personal health record.
8. I am also happy for information about me related to the study being stored on a password protected computer system, which will be backed-up in a separate location to keep this information safe

| Participant’s signature  (or thumbprint) | Print name | Date | | | | | | | |
| --- | --- | --- | --- | --- | --- | --- | --- | --- | --- |
|  |  | *D* | *D* | *M* | *M* | **2** | **0** | *Y* | *Y* |

| Witness’s signature  (if thumbprint used above) | Print name | Date | | | | | | | |
| --- | --- | --- | --- | --- | --- | --- | --- | --- | --- |
|  |  | *D* | *D* | *M* | *M* | **2** | **0** | *Y* | *Y* |

| Midwife’s/ Doctor’s signature | Print name | Date | | | | | | | |
| --- | --- | --- | --- | --- | --- | --- | --- | --- | --- |
|  |  | *D* | *D* | *M* | *M* | **2** | **0** | *Y* | *Y* |

When completed 1 form for participant, 1 form for researcher/site file, 1 form for medical notes
