# Supplementary material for: Prophylactic antibiotics to reduce pelvic infection in women having miscarriage surgery – The AIMS (Antibiotics in Miscarriage Surgery) trial: study protocol for a randomized controlled trial
Source: Trials. 2018 Apr 23;19:245. doi: 10.1186/s13063-018-2598-3 (PMC5914072; doi:10.1186/s13063-018-2598-3)
Supplement: Supplementary file 3 — Patient information sheet. (DOCX 65 kb) [file 13063_2018_2598_MOESM3_ESM.docx]

# Additional file 3

Local Logo

# Patient information sheet


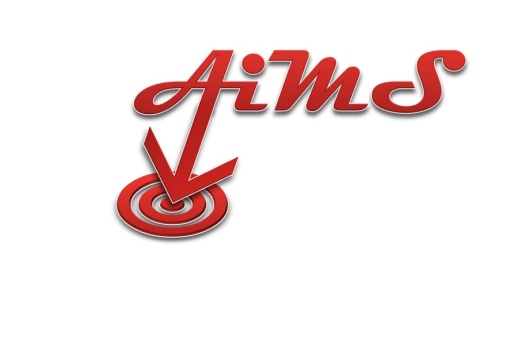


**AIMS – Antibiotics in miscarriage surgery**

**Participant Information Sheet**

We would like to invite you to take part in a research study. Whether you take part or not is entirely your choice. Before you decide, you need to understand why the research is being done and what it would involve for you.

We want to see whether the use of antibiotics prior to miscarriage surgery reduces the chance of infection following surgery. This study is called the AIMS (Antibiotics In Miscarriage Surgery) Trial.

Please ask us if there is anything that is not clear or if you would like more information.

**SUMMARY**

Infections of the pelvis following miscarriage surgery can be serious and also lead to long-term health problems.

For some types of surgery we know that antibiotics used before the operation can reduce these risks. However, nobody knows if this will help for miscarriage surgery and therefore this is an important question that the AIMS trial hopes to answer.

We are asking women who need miscarriage surgery to take part in this study. Half the women will be given a single dose of 2 antibiotics (Metronidazole and Doxycycline) 2 hours before surgery and half the women will be given dummy tablets. Dummy tablets look like the real antibiotics but they don’t have any active ingredients.

If you take part, we would then like to see how you are for the two weeks after surgery. We will ask you to return for an appointment 2 weeks after surgery, but we will also ask you to come to the hospital or telephone us if you notice any problems at all before this appointment.

[
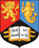
](http://www.bham.ac.uk/)
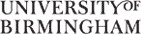
[
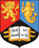
](http://www.bham.ac.uk/)
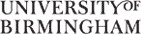


This research is sponsored by the University of Birmingham, United Kingdom. The research has been funded by the Medical Research Council, United Kingdom, the Wellcome Trust and the Department for International Development, United Kingdom.

This research is sponsored by The University of Birmingham, United Kingdom. The research has been funded by the Medical Research Council, United Kingdom, the Wellcome Trust and The Department for International Development, United Kingdom.

This research is sponsored by The University of Birmingham, United Kingdom. The research has been funded by the Medical Research Council, United Kingdom, the Wellcome Trust and The Department for International Development, United Kingdom.

This research is sponsored by The University of Birmingham, United Kingdom. The research has been funded by the Medical Research Council, United Kingdom, the Wellcome Trust and The Department for International Development, United Kingdom.

**What is the purpose of the study?**

The purpose of this study is to find out whether treating women with antibiotics before they have miscarriage surgery reduces the risk of infection following surgery. The antibiotics are Doxycycline 400mg and Metronidazole 400mg.

**Why have I been invited?**

You have been invited to take part in the study as you are going to have miscarriage surgery.

**Do I have to take part?**

No. It is up to you whether or not you take part. If you wish to take part, you will be given this information sheet to keep and will be asked to sign a consent form. You are still free to withdraw at any time and without giving a reason. If you decide not to take part or decide to withdraw at any time, that will not affect your medical care in any way.

**What will happen to me if I take part?**

If you decide to take part in the study, and have signed the consent form, the researcher will ask you to take the trial medication 2 hours before the surgery. The antibiotics and the dummy tablets look exactly the same, so neither you nor the researcher will know which you have received. There will be a total of 5 tablets that you will be asked to swallow with water.

You will then have the surgery as planned (unless there is some other reason for not doing it). Taking part in the trial will not make any difference to your operation.

After the surgery, it is very important to let the doctor or nurse know if you have any problems at all. If you have any problems at any time before you go home, please tell the doctor or nurse looking after you. If you have any problems during the first two weeks after you leave hospital, please contact us and we will see you as soon as possible. We’ve put our telephone number at the bottom of this page.

Even if you are well, we ask you to come back 2 weeks after the surgery to have a check-up. When you do, or if you return at another time with problems after the surgery, we will do a blood test to check for signs of infection.

If you have an infection we will do some more tests to find out the cause of this infection and to make sure that you get the right treatment.

**What should I do after the surgery?**

Please let us know if you notice any of these things at any time in the 14 days after surgery:

- Pain in your lower abdomen (belly)
- Feeling feverish, hot and cold or shivery
- Any unusual discharge from the vagina, especially if it has a bad smell
- Bleeding from the vagina gets heavier or you pass big blood clots
- Feeling generally unwell
- Any rashes or swelling
- Any other things that worry you

This information can also be found on the “Follow-up card”

**What is the drug being tested?**

The drugs being tested are Doxycycline (400mg) and Metronidazole (400mg). These are already used for the treatment of pelvic infections and before other types of surgery. They are normally effective and do not normally have serious side-effects.

**What are the possible disadvantages and risks of taking part?**

You might have some small side-effects from Doxycycline or Metronidazole, but they don’t happen often. If you notice any problem that you think may be because of the treatment, please let the doctor or nurse that treated you know as soon as possible.

**What are the possible benefits of taking part?**

We do not know if the study will help you personally, but the information we will get may help improve the care for women in the future.

If you are taking part in the study we will follow your health very carefully after the surgery and will help you to receive prompt treatment if there are any problems.

**What if there is a problem? What if something goes wrong?**

***Complaints***

If you have a concern about any part of this study, you should ask to speak to the local research nurse or doctor, who will do their best to answer your questions. We have put their contact details at the end of this information sheet. If you are still unhappy after talking to them and you want to complain formally, you can do this. You can either contact the local research ethics committee who have given us permission to conduct this study, or you can contact the central trial management team. You can get their details from the local doctor or nurse that treated you. You have the same rights whether or not you take part in this study.

***Harm***

University of Birmingham and further local insurers hold insurance policies which apply to this study. If you experience harm or injury as a result of taking part in this study, you may be eligible to claim compensation.

If you are harmed due to someone’s negligence, then you may have grounds for a legal action.

Please contact the local research lead if you would like further information on this.

**Will my taking part in this study be kept confidential?**Yes. All information collected from you for the purposes of this study will be kept strictly private and confidential.

Any information that is passed outside the hospital or the University of Birmingham will have any identifying details like your name, age and address removed so that the information is and stays completely anonymous. All information will be held securely and in strict confidence. To ensure the safety of all the information gathered, electronic back-up copies will be stored remotely on encrypted, secure password protected databases.

You will not be identified personally in any publication of results from this study.

Sometimes the research information is inspected, for example to make sure that all the women taking part in the study have agreed to do so. But apart from this, only study organisers will be able to see personal details.

**What will happen to the results of the research study?**

When the results of the AIMS study are known, we will talk about it at a local meeting. We will also publish the results of the study in medical journal(s). We will also make the information available on our website ([www.aimstrial.org](http://www.aimstrial.org)) for the general public.

**Who has reviewed the study?**

This study has been reviewed and approved by the Ethical clearance committee of [LOCAL COMMITTEE TO BE INSERTED]. This is an independent group of people who are responsible for making sure that studies meet the required standard and that patients have their safety, rights, wellbeing and dignity protected.

**Do you have any further questions?**

Once you have read this leaflet and discussed it with the researcher, we hope that you will choose to take part in the AIMS study. If you have any questions about the study now or later, please feel free to ask us. The contact details are found at the end of this leaflet.

You will be given a copy of the information sheet and a signed consent form to keep.

Thank you for taking time to read this sheet and for considering taking part in the study.

**How to contact us:**

Please phone us at any time, day or night, (on the study telephone study number to be inserted) on [local number to be inserted].

A member of study staff can also be found during the day at [local number to be inserted].

We will provide you with telephone credit so it is easier to contact us if you have any problems or would like to ask any questions.

We will pay your travel costs to the hospital when you come back after two weeks and also if you need to come back before then.

We will contact you to check that you don’t have any problems and to remind you about the follow-up appointment. We will also contact you if you don’t attend your follow-up appointment, to check that everything is OK. We will find out from you how you would like to be contacted.

**Further Contact Details:**

Name:_______________________________ Phone Number: __________________________

**Thank you for taking time to read this sheet and for considering taking part in the study.**
